# Advances in Polymer Film and Coating Technologies for Enhanced Surface Functionality

**DOI:** 10.3390/polym18080918

**Published:** 2026-04-09

**Authors:** Rashid Dallaev

**Affiliations:** Department of Physics, Faculty of Electrical Engineering and Communication, Brno University of Technology, Technická 2848/8, 61600 Brno, Czech Republic; rashid.dallaev@vut.cz

**Keywords:** polymer coatings, surface modification, plasma treatment, plasma polymerization, self-cleaning surfaces, photocatalytic coatings, superhydrophobicity, antimicrobial coatings, corrosion protection, sustainable coatings

## Abstract

Polymer films and coatings play an increasingly critical role in extending material functionality across industrial, biomedical, and environmental applications. Recent advances in surface engineering have enabled precise control of interfacial properties, leading to enhanced durability, cleanliness, and protection. This review summarizes state-of-the-art strategies for modifying polymer surfaces, with an emphasis on plasma-based surface modification and plasma-induced polymerization as versatile, solvent-free methods for tailoring wettability, chemical functionality, and adhesion. Furthermore, it examines emerging classes of self-cleaning and self-sterilizing coatings that leverage photocatalytic, hydrophobic, or antimicrobial mechanisms to mitigate contamination, biofouling, and pathogen transmission. Additionally, developments in high-performance barrier films designed to protect food products and electronic devices through improved resistance to gases, moisture, and chemical agents are highlighted. By integrating insights from materials chemistry, surface physics, and nanostructured coating design, this review provides a comprehensive overview of current achievements and future directions in functional polymer films and coatings aimed at anti-pollution, antibacterial, and anti-corrosion performance.

## 1. Introduction

Polymer films and coatings are ubiquitous in modern technology, serving as protective or functional layers across industries from packaging and electronics to biomedicine and environmental engineering [1,2]. These thin polymeric layers impart desirable surface properties—for example, they can enhance abrasion and corrosion resistance on metals, provide semi-permeable barriers in food packaging, or tailor wettability and adhesion for biomedical devices. In food processing, edible polymer coatings (e.g., based on proteins, polysaccharides, or lipids) form semi-permeable films that slow moisture and gas exchange, dramatically extending shelf life [3,4]. On steel and alloys, polymeric coatings (epoxies, polyurethanes, silicones, etc.) provide a robust moisture and chemical barrier while also imparting corrosion resistance and surface finish [5]. In electronics and optics, transparent polymer barriers protect sensitive components from oxygen, moisture, and particulates, often requiring added functionalities (e.g., flexibility, self-healing) for foldable or wearable devices [6].

Because unmodified polymers often have mismatched surface energies or bio-incompatible interfaces, considerable research focuses on surface engineering to tailor interfacial properties. Broadly, surface modification strategies fall into two classes: physical treatments (e.g., plasma, corona discharge, UV/ozone, ion beams) and chemical grafting or deposition (e.g., chemical vapor deposition, self-assembled monolayers, wet chemistry). These techniques introduce new functional groups or textures to the polymer surface to adjust wettability, adhesion, and chemical functionality [7,8]. For instance, treating a polymer with a flame or corona discharge can increase surface energy by oxidizing the surface, while deposition of a thin primer or adhesion layer can chemically bridge hydrophobic polymers to polar coatings. Notably, plasma-based methods have emerged as powerful, solvent-free tools for polymer surface modification. Non-equilibrium (cold) plasmas generate energetic ions, electrons and radicals that can ablate, cross-link, or graft new moieties on the surface [9,10]. Primc et al. note that plasma treatments can substantially increase a polymer’s surface free energy (i.e., wettability), which often correlates with improved bonding of subsequent coatings [7]. In practice, gaseous plasma immersion or dielectric barrier discharges can introduce oxygen- or nitrogen-containing polar groups (-OH, -COOH, -NH_x_) or deposit ultra-thin polymer films directly from monomer vapor (plasma polymerization) [11,12,13]. Because these methods operate in vacuum or controlled atmospheres, they avoid solvents and allow conformal coverage over complex geometries.

**Figure 1:**
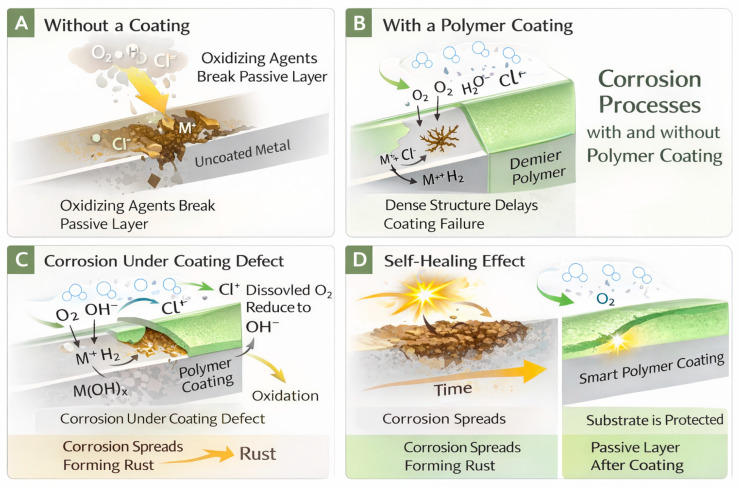
Schematic illustration of corrosion processes and the role of polymer coatings, presented as subdivided panels for clarity. (**A**) Corrosion initiation on uncoated metal: oxidizing species (O_2_, H_2_O, Cl^−^) penetrate and disrupt the passive layer, leading to metal oxidation (M → M^x+^). (**B**) Intact polymer coating: the dense barrier limits the ingress of corrosive species, thereby slowing electrochemical reactions and delaying corrosion initiation. (**C**) Localized corrosion under coating defects: microcracks or pores allow the diffusion of oxygen, water, and ions to the substrate, promoting electrochemical reactions and the formation of corrosion products (e.g., M(OH)_x_), which propagate laterally. (**D**) Long-term behavior and mitigation: corrosion spreads over time in defective regions, whereas advanced (e.g., self-healing) coatings can restore barrier properties and re-form a protective layer, improving substrate durability. Reproduced from [3] (The figure is available Open Access).

Protective coatings mitigate corrosion by forming a passive barrier that limits the penetration of water and ions (Figure 1, right). A more compact and dense structure slows the diffusion of corrosive species, thereby reducing oxidative degradation and enabling long-term protection-even with coatings only a few micrometers thick. Despite their effectiveness, even high-quality coatings can absorb some moisture when exposed to aqueous or electrolyte environments. Under such conditions, strong adhesion between the coating and the underlying substrate becomes essential to prevent delamination, which would otherwise accelerate corrosion processes. When water or electrolytes infiltrate defects in the coating or reach the metal surface, advanced “smart” coatings can provide additional protection. These systems incorporate corrosion inhibitors or self-healing components that can modify interfacial charge transfer, promote the formation of protective passive layers, or repair the barrier itself, thereby enhancing durability and prolonging the coating’s service life [3].

### 1.1. Relevancy

The development of advanced polymer films and coatings is highly relevant in the context of current global technological, environmental, and public health challenges. Modern industries increasingly require materials that not only provide passive protection but also exhibit active and multifunctional surface properties. In sectors such as healthcare, food packaging, electronics, and infrastructure, there is a growing demand for coatings that can simultaneously enhance durability, prevent contamination, and reduce maintenance costs. The COVID-19 pandemic further highlighted the importance of antimicrobial and self-sterilizing surfaces, accelerating research into coatings capable of limiting pathogen transmission on high-contact surfaces [14,15].

At the same time, sustainability considerations are driving the transition toward environmentally friendly and solvent-free processing methods. Plasma-based surface modification techniques, as discussed in this review, offer a compelling solution by enabling precise control of surface chemistry without the use of hazardous chemicals. These approaches align with stricter environmental regulations and the broader push toward green manufacturing. Additionally, the increasing use of flexible electronics, biodegradable packaging, and smart materials underscores the need for coatings that can be tailored at the nanoscale while maintaining mechanical integrity and functionality [16,17].

This review is therefore timely in providing a comprehensive overview of recent advances in polymer coating technologies, particularly in the areas of self-cleaning, antimicrobial, and barrier functionalities. By integrating developments in surface engineering, nanostructured materials, and plasma processing, the paper addresses key scientific and industrial challenges. It also highlights emerging trends toward multifunctional and sustainable coatings, offering valuable insights for researchers and engineers working to design next-generation materials with enhanced performance and reduced environmental impact.

### 1.2. Methodology

This review is based on a comprehensive analysis of a large body of peer-reviewed scientific literature focusing on polymer films and coating technologies. An extensive collection of sources was gathered from major academic databases (Web of Science; Scopus; Google Scholar; PubMed and others), with particular emphasis on studies published in recent years to ensure that the discussion reflects the latest advancements in the field. Priority was given to high-impact journal articles, review papers, and experimental studies addressing surface modification techniques, self-cleaning and antimicrobial coatings, barrier materials, and plasma-based processing methods.

The selected literature was systematically evaluated and categorized according to key thematic areas, including photocatalytic, hydrophobic, and antimicrobial coatings, as well as plasma-assisted surface engineering strategies. By synthesizing findings from a broad and up-to-date range of sources, this methodology enables a coherent and well-supported assessment of recent progress, emerging trends, and future directions in functional polymer coating technologies.

## 2. Emerging Self-Cleaning and Self-Sterilizing Surface Coatings

Microbial contamination and biofouling on surfaces pose serious health and economic challenges. Pathogens such as bacteria and viruses can persist on surfaces for days at infectious concentrations [18], enabling indirect “fomite” transmission of disease. Self-cleaning and self-sterilizing coatings are being developed to passively reduce this risk. Broadly, coatings fall into passive anti-fouling types (reducing adhesion) and active biocidal types (killing microbes) [19,20]. Passive coatings often employ engineered surface wettability (e.g., hydrophobicity or superhydrophobicity) to shed water and dirt, whereas active coatings incorporate biocidal agents (metals or photocatalysts) to inactivate organisms [21,22]. This review covers three emerging classes of such coatings—photocatalytic, hydrophobic/superhydrophobic, and active antimicrobial—comparing their mechanisms, materials, fabrication, and performance across diverse applications (healthcare, public surfaces, marine, packaging, construction, etc.). Further the review emphasizes developments of the past decade, and highlights how combinations of these strategies can yield multifunctional “super-clean” surfaces. Each scientific point is supported by recent peer-reviewed literature.

A major thrust in advanced coatings is enabling active surface hygiene and cleanliness. Self-cleaning surfaces leverage two main strategies: (1) inducing superhydrophobicity so that water droplets roll off and sweep away contaminants (“lotus effect”), or (2) using photocatalysis to chemically degrade organics. In hydrophobic self-cleaning coatings, engineered micro/nanostructures combined with low-surface-energy chemistry yield extremely high water contact angles (often >150°) and very low sliding angles [23,24]. For example, lotus-leaf mimetic surfaces exhibit contact angles above 160°; falling droplets bead up and roll off, entrapping dust and dirt along the way. The lotus leaf is a benchmark: its dual-scale roughness leads to water sliding that continuously clears particles, so the plant stays clean in muddy water [24]. Artificial superhydrophobic polymer composites (often incorporating silica or fluorinated nanoparticles) achieve similar behavior, maintaining clean surfaces in outdoor and industrial conditions. Crucially, such rough hydrophobic coatings must also be mechanically robust—recent work integrates hard nanoparticles or cross-linked siloxanes to retain high hardness and wear resistance while preserving the lotus effect [25,26].

Hydrophilic self-cleaning coatings rely instead on photo- or electro-catalysis. In this category, semiconductor materials (especially TiO_2_) are embedded in a polymer matrix or deposited as a thin film. Under UV or visible light, TiO_2_ generates reactive oxygen species that break down organic films and pollutants on the surface [27]. Importantly, these coatings often become superhydrophilic after activation (wetting spontaneously), so that rainwater spreads into a film and washes away the degraded residues. Thus, photocatalytic coatings can in principle continuously decompose oils or microbial films: after irradiation, any remaining dirt is washed off easily by water. This photocatalytic self-cleaning is inherently solvent-free and can operate in daylight, but in practice requires UV exposure and maintenance of catalyst activity. For transparent applications (e.g., solar panel covers, windows), researchers have developed TiO_2_-SiO_2_ multilayer films that combine anti-reflection with photocatalysis [28,29,30]. These films degrade environmental pollutants (like methyl orange or phenol) under illumination while remaining optically clear; for instance, one study showed a TiO_2_-SiO_2_ coating decomposed ~30% of phenolic contaminants after 12 h of UV exposure [23].

**Figure 2:**
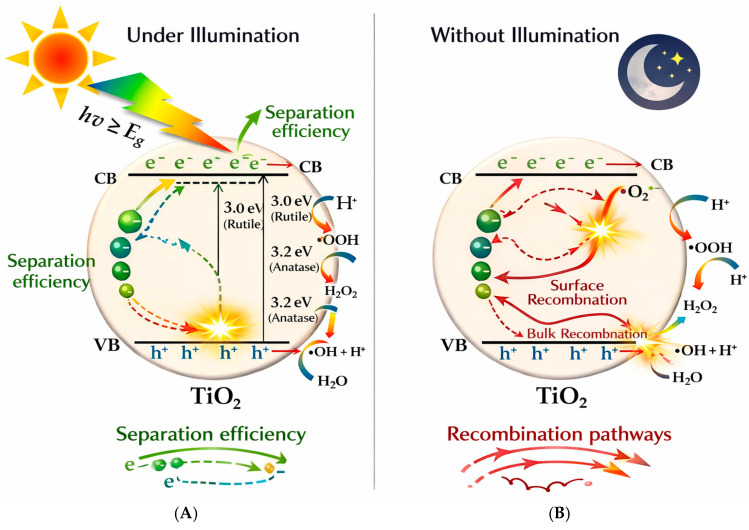
Schematic representation of charge carrier dynamics and reactive species generation in TiO_2_ under illuminated and non-illuminated conditions. (**A**) Under illumination (hν ≥ E_g_): incident photons excite electrons from the valence band (VB) to the conduction band (CB), generating electron–hole (e^−^/h^+^) pairs. Efficient charge separation enables photogenerated electrons to reduce O_2_ into reactive oxygen species (e.g., •O_2_^−^, •OOH, H_2_O_2_), while holes oxidize H_2_O to produce •OH radicals. Competing recombination pathways are also indicated, highlighting their impact on overall photocatalytic efficiency. (**B**) Without illumination: in the absence of photoexcitation, no electron–hole pairs are generated, and recombination dominates. Surface and bulk recombination pathways suppress charge carrier availability, preventing the formation of reactive oxygen species and limiting photocatalytic activity. Reproduced from [29] (The figure is available Open Access).

Figure 2 depicts the TiO_2_ photocatalytic mechanism in which highly reactive oxidizing species are generated. The bandgap energies (Eg) are approximately 3.0 eV for the rutile phase and 3.2 eV for the anatase phase. When TiO_2_ is exposed to light with energy equal to or greater than its bandgap (hv ≥ Eg), it absorbs photons, promoting electrons from the valence band (VB) to the conduction band (CB). This excitation produces free electrons (e^−^) and corresponding positively charged holes (h^+^).

These charge carriers exhibit distinct reactivity: the electrons act as reducing agents, while the holes function as strong oxidants. After migrating to the TiO_2_ surface, they participate in redox reactions with adsorbed oxygen and water molecules. Specifically, electrons reduce oxygen to generate superoxide radicals (O_2_•^−^), while holes oxidize water to produce hydroxyl radicals (•OH). Additional reactive oxygen species, including hydroperoxide radicals (•OOH) and hydrogen peroxide (H_2_O_2_), may subsequently form through further redox reactions, as well as through processes such as dimerization and disproportionation during photocatalysis

Polymers have also been engineered to be nonfouling via steric repulsion: hydrophilic, brush-like polymers (e.g., polyethylene glycol, poly(oxazoline), zwitterionic polymers) resist protein and cell adhesion by holding a tightly bound water layer [31,32]. Zwitterionic polymer brushes in particular have shown up to ~99% reduction in bacterial attachment in laboratory tests [1]. Combining approaches is common; for example, Zhang et al. reported an epoxy-siloxane hybrid coating containing both a lubricating polydimethylsiloxane (PDMS) phase and antifouling telomer additives, yielding a transparent coating with ceramic-like hardness that also resisted biofilm formation [33]. In food and medical settings, polymer–metal composite coatings are used: Ag or Cu nanoparticles can be dispersed in polyurethane or epoxy matrices to kill pathogens, while fluorinated or silicone topcoats provide cleanability [34,35]. Overall, antimicrobial polymer coatings are now widely deployed on medical implants, food processing equipment, and public surfaces—a trend driven by safety and regulatory demands [36,37].

**Figure 3:**
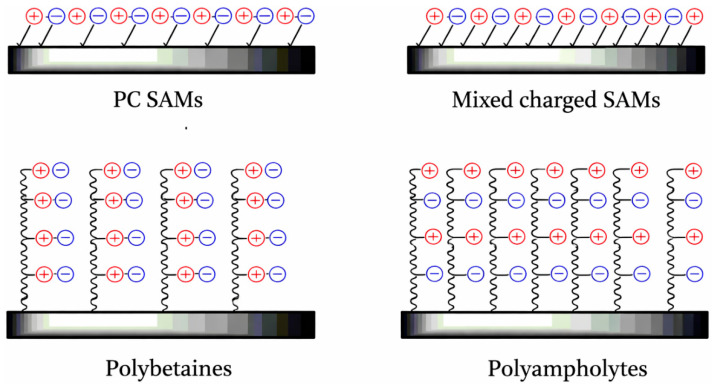
Schematic representation of the design principles for developing nonfouling polyzwitterionic materials. These surfaces are characterized by a uniform distribution of charges and an overall balance between oppositely charged groups, enabling them to exhibit strong resistance to protein adsorption [31]. (The figure is available Open Access).

Zwitterionic materials are widely regarded as biocompatible due to their biomimetic nature. Nonfouling polyzwitterions can generally be divided into two categories: polybetaines, which contain both positive and negative charges within the same monomer unit, and polyampholytes, where these oppositely charged groups are located on separate monomer units. A key aspect governing their resistance to fouling is the precise control of charge balance and the uniform distribution of positive and negative groups across the surface (Figure 3). This principle serves as an important framework for developing new nonfouling polyzwitterionic systems. By maintaining such balance, these materials enhance surface hydration while minimizing electrostatic interactions with proteins. The following section highlights recent progress in designing polybetaines and polyampholytes for biological surface applications based on this concept [31].

### 2.1. Photocatalytic Self-Cleaning Coatings

Photocatalytic coatings exploit light-activated semiconductor materials (e.g., TiO_2_, ZnO, WO_3_, g-C_3_N_4_, metal-doped oxides) to generate reactive oxygen species (ROS) that oxidize organic contaminants and kill microbes. Under UV or visible irradiation an electron is excited from the semiconductor’s valence band into its conduction band, creating an electron-hole pair; the hole reacts with surface water or hydroxide to form hydroxyl radicals (•OH) while the electron reduces oxygen to superoxide radicals (O_2_•^−^) [38,39,40,41]. These ROS (•OH, H_2_O_2_, singlet O_2_, etc.) are highly oxidative, damaging microbial membranes, DNA, proteins and biofilm matrices. In fact, a recent meta-analysis found that TiO_2_ coatings reduced both Gram-positive and Gram-negative bacterial counts by over 99% [38]. Photocatalysis thus provides a self-regenerating “sunlight-sanitizing” mechanism: as long as light is present, new ROS are continuously produced.

Common photocatalytic materials include nano-structured TiO_2_ and ZnO. However, these are limited to UV light. Recent advances focus on band-gap engineering and heterojunctions to extend activity into the visible spectrum [42,43]. Doping with transition metals (Cu, Fe, N, etc.) or coupling TiO_2_ with narrow-bandgap semiconductors (e.g., CdS, g-C_3_N_4_) or carbonaceous supports can enhance visible-light absorption and reduce electron-hole recombination [44,45,46]. For example, Cu-doped ZnO films exhibited improved visible-light photocatalytic degradation and bacterial inactivation compared to pure ZnO [47,48]. Photocatalysts are typically immobilized onto substrates by sol–gel or spray coatings, sputtering or CVD, or by embedding oxide nanoparticles in a binder layer [49,50]. Fabrication methods range from simple dip- or spray-coating of pre-formed particles, to in situ growth (e.g., hydrothermal, plasma-assisted) of nanostructured films.

In study [49], the authors describe a straightforward and environmentally friendly method for producing photocatalytic coatings composed of titanium dioxide and geopolymers, notably without the need for any heat-based post-treatment. A mixture of P25 titania powder, potassium silicate, and a calcium aluminate hardening agent was prepared in water and deposited onto aluminum substrates using three techniques: brushing, rolling, and spray application. The coated samples were then left to dry in air for 12 h.

**Figure 4:**
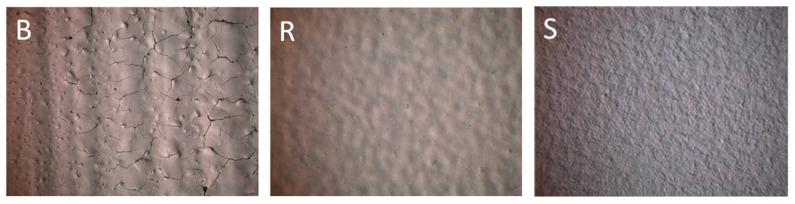
Optical microscope images showing the surface morphology of coatings applied using different techniques (B, R, and S) [49]. (The figure is available Open Access).

Optical microscopy images presented in Figure 4 highlight clear distinctions among the coatings produced by these methods (brush-B, roller-R, spray-S), following an additional 24 h of air drying. The brush-applied coating, identifiable by vertical stroke patterns, exhibited uneven thickness, poor adhesion to the substrate, high porosity, and extensive cracking. These defects are attributed to thickness variations, which caused irregular drying and promoted crack formation in thicker regions. Given these surface inconsistencies and the insufficient adhesion, the brush application method was excluded from further analysis [49].

Samples R and S both exhibit a consistent and uniform surface morphology, with no observable cracks or structural imperfections. However, sample S appears to possess a slightly smoother finish compared to sample R. Based on these characteristics, both preparation methods (R and S) were deemed appropriate for the deposition of catalytic coatings.

**Figure 5:**
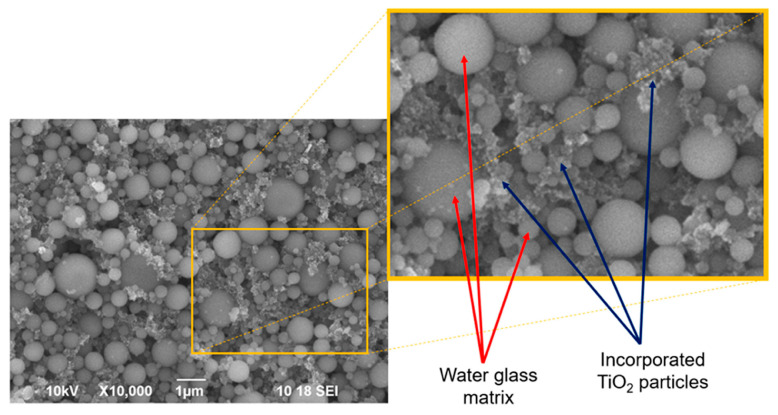
SEM micrographs depicting the nanostructured surface morphology of sample R [49]. (The figure is available Open Access).

High-resolution SEM micrographs presented in Figure 5 reveal the detailed surface morphology of coating 2-R. Clusters of nanoscale TiO_2_ particles are dispersed within the gaps between the rounded geopolymer matrix grains. The hardened composite structure shows a notably porous nature. The morphology of coating 2-S (image not included) is reported to be nearly indistinguishable from that of 2-R.

Performance of photocatalytic coatings is evaluated by both pollutant degradation tests (dye or VOC breakdown) and direct microbial assays. Standard microbiological tests (e.g., CFU counting after defined light exposure, ISO 22196) show substantial kill rates. For instance, one report noted complete killing (6 log reduction) of *E. coli* and *S. aureus* on illuminated TiO_2_/Ti surfaces within a few hours [51,52,53,54]. Antiviral efficacy has also been shown: a daylight-illuminated TiO_2_ coating on glass inactivated a coronavirus model by >99% after several hours [34]. However, efficacy depends on light intensity and wavelength; many conventional TiO_2_ coatings require UV (~365 nm) to be effective. Ongoing work seeks visible-light catalysts so surfaces can sterilize under indoor lighting [55,56]. Recent implant-focused studies illustrate this trend: “visible-light-triggered” photocatalytic implants have been engineered that generate ROS under dental-operatory or ambient light, offering on-demand disinfection of implant surfaces [57,58,59].

Photocatalytic coatings have been deployed in applications like self-cleaning glass, air purification and water treatment. For example, TiO_2_-coated architectural glass (Pilkington Activ, Sto NCS, etc.) degrades organic dirt and maintains clear surfaces. In healthcare settings, UV-activated coatings on hospital surfaces have shown reduced microbial loads under lighting [38,60,61]. For water treatment, thin-film photocatalytic membranes can purify water while resisting biofilm formation. Notably, one study achieved >99% inactivation of antibiotic-resistant bacteria and degradation of their resistance genes under UV LED with a TiO_2_ photoreactor [62,63]. Advantages of photocatalysts include continual disinfection without added chemicals, and simultaneous removal of organic pollutants. Key limitations are the need for a light source (especially UV), potential generation of harmful by-products (e.g., ozone or partial oxidation products), and catalyst deactivation or leaching over time. Advances in low-energy activation and robust immobilization aim to address these issues [64,65,66].

### 2.2. Hydrophobic and Superhydrophobic Coatings

Hydrophobic coatings, and particularly superhydrophobic ones, provide a passive self-cleaning mechanism by repelling water and thus carrying away surface contaminants. Superhydrophobic surfaces are defined by a water contact angle > 150° and rolling/sliding angle < 10°, achieved through a combination of micro-/nano-scale roughness and low-surface-energy chemistry (e.g., fluorosilanes, hydrocarbon waxes). These features trap air beneath water droplets (Cassie–Baxter state), causing water to bead up and roll off easily. The Cassie–Baxter state refers to a situation where a liquid droplet sits partly on a solid surface and partly on trapped air pockets within its micro-/nano-scale roughness. Because the droplet is not in full contact with the surface, adhesion is very low. As a result, water forms nearly spherical droplets that easily roll off, picking up dirt and microbes along the way—this is what enables the strong self-cleaning (“lotus effect”) behavior of superhydrophobic coatings. As droplets roll, they collect and remove dirt, organic debris, and loosely adhered microbes—the classic “lotus-leaf effect”. In nature this is seen on lotus leaves, insect wings, etc., and in the lab many hierarchical textured coatings mimic this [67].

Superhydrophobic coatings have demonstrated strong anti-fouling properties. By repelling water, these surfaces greatly reduce the contact area between microbes and the substrate. Research shows that superhydrophobic biomimetic surfaces can inhibit bacterial adhesion and limit biofilm formation [68,69]. For instance, mosquito- or termite-wing-like nanostructures have been shown to resist colonization by bacteria. In one marine study, a superhydrophobic ZnO nanowire/silane-treated steel surface (contact angle ~152°) achieved >90% reduction in bacterial adhesion compared to uncoated steel. Similarly, a silane-modified titanium surface became superhydrophobic and reduced bacterial cell attachment by over 90% [70]. Such coatings thereby offer continuous self-cleaning without biocides.

However, purely hydrophobic surfaces are passive—they prevent microbes from sticking but do not actively kill those that remain on the surface. As noted by Zhao et al., superhydrophobicity provides anti-adhesion and self-cleaning effects but cannot sterilize by itself [67]. To achieve true antimicrobial action, researchers often incorporate biocidal agents into or onto the hydrophobic matrix (for example embedding Ag, Cu or TiO_2_ nanoparticles, or tethering cationic polymers). The synergistic combination yields both repulsion and inactivation. A recent example is an ACS Omega report of CuO-TiO_2_ hybrid coatings on plastic: these films were superhydrophobic (water contact angle > 150°) and at the same time exhibited complete bacterial kill (no detectable *E. coli* or *S. aureus* after 24 h) [71]. The embedded CuO supplied a strong antimicrobial action while the TiO_2_ provided durability and adhesion of the coating.

Hydrophobic coatings find many practical uses. In construction, “self-cleaning” façade paints, roof tiles, and windows keep building exteriors cleaner [23]. Outdoor applications (solar panels, signs) use hydrophobic glass treatments to shed rainwater and dust [72]. In textiles and apparel, durable hydrophobic treatments repel spills and prevent staining [73]. In marine engineering, hydrophobic paints can reduce drag and mitigate fouling (though true superhydrophobic surfaces underwater are challenging, the lotus-inspired plastron effect has been explored) [74,75,76]. Healthcare textiles (e.g., bed linens, gowns) and consumer products (e.g., touchscreens) increasingly employ nano/micro-structured hydrophobic coatings for easier cleaning [77]. Performance is usually quantified by static contact angle, sliding angle, and durability tests (abrasion, tape-peel, UV exposure). Many reported coatings can survive thousands of abrasion cycles or strong solvents, but a general limitation is that superhydrophobicity often degrades over time or under harsh wear. Furthermore, many superhydrophobic coatings rely on fluorinated compounds (e.g., perfluoroalkyl silanes), raising environmental concerns. The field is moving toward fluorine-free and bio-inspired chemistries [78,79,80].

**Figure 6:**
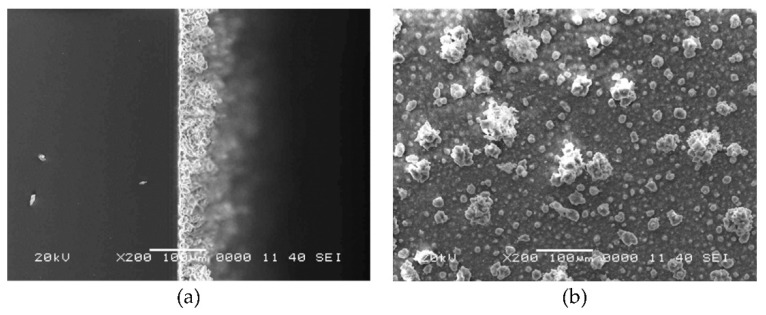
Scanning electron microsy (SEM) images illustrating both the surface morphology and cross-sectional structure of a coating composed of TSR194 resin and octyl-functionalized colloidal silica: (**a**) cross-sectional view; (**b**) surface view [80]. (The figure is available Open Access).

In study [80], the authors describe a coating system composed of epoxy-modified silicone resin (TSR194) combined with fluorinated colloidal silica, which exhibits strong superhydrophobic behavior along with good structural integrity. To develop a fluorine-free alternative, the researchers modified colloidal silica particles (340 nm in size) using octyl chlorosilane, applying the same surface treatment approach. These octyl-functionalized silica particles were then blended with the TSR194 resin, with the particle content adjusted to 60% by volume. The resulting superhydrophobic coating was fabricated through a spray application followed by thermal curing. Representative SEM images of both the surface morphology and cross-sectional structure are presented in Figure 6.

### 2.3. Active Antimicrobial Coatings

Complementing self-cleaning, self-sterilizing (antimicrobial) polymer coatings are an active research frontier, especially given public health concerns. Such coatings inhibit bacterial or viral attachment, thereby reducing biofouling and pathogen transmission on surfaces. Strategies fall into two broad types [81]:

- Release-based coatings embed biocidal agents (silver nanoparticles, copper ions, antibiotics, essential oils, etc.) that gradually leach out, creating a local antimicrobial environment. These can be very effective initially—for example, AgNP-filled polymers can kill > 99% of common bacteria (e.g., *Escherichia coli*, *Staphylococcus aureus*, and *Pseudomonas aeruginosa*)—but they suffer limited lifetime once the agent is depleted [82,83,84].
- Contact-active coatings immobilize antimicrobial groups directly onto the polymer surface. For example, quaternary ammonium salts or covalently bound metal oxides eliminate microbes upon contact without releasing chemicals. These surfaces can remain active indefinitely and minimize environmental release of biocides, but they require sufficient exposure of active sites to function effectively [85,86,87,88].

Active antimicrobial coatings kill or inactivate microbes upon contact. Two main strategies are widely used: (1) biocide-releasing materials, typically metal or metal-oxide nanoparticles embedded in a binder that slowly release toxic ions; and (2) contact-killing surfaces, often with immobilized biocides (e.g., quaternary ammonium polymers, antimicrobial peptides) or nanostructures that mechanically rupture cells. Common antimicrobial agents include silver (Ag), copper (Cu), zinc (Zn), and TiO_2_ (with or without light), as well as newer agents like graphene derivatives or antimicrobial polymers [89,90].

Metal-based coatings are among the most prevalent. Silver ions and nanoparticles are well-known broad-spectrum antimicrobials that disrupt bacterial membranes and denature proteins. Copper and copper alloys (brasses, bronzes) also kill bacteria and many viruses by generating oxidative stress and damaging envelope structures. For example, high-touch copper surfaces (doorknobs, rails) have been shown to kill coronaviruses rapidly. In one study, SARS-CoV-2 survived only ~4 h on copper surfaces, versus days on stainless steel or plastic [91,92]. Similarly, surfaces impregnated with Ag or ZnO nanoparticles can achieve rapid bacterial kill. These metal/metal-oxide coatings have been commercialized for doors, hospital equipment, air ducts, face masks, etc. Notably, Martín et al. reported that Cu, Ag, Zn, and TiO_2_ coatings on high-touch items (facemasks, trolleys, door handles, handrails) significantly reduce microbial reservoirs. Such coatings act continuously without user intervention, forming a passive barrier against contamination [93,94].

Nanostructured surfaces form another active strategy. Inspired by insect wings, one can etch high-aspect-ratio nanopillars into surfaces that physically pierce or stretch bacterial envelopes, causing death [95]. For example, titanium nanotextures modeled on dragonfly wings have been shown to deform *E. coli* cells on contact. In practice, combining nanostructuring with biocidal coatings gives “binary killing” surfaces. Birkett et al. highlight that contact-killing, nanoprotrusion, and superhydrophobicity phenomena can be merged into multi-functional coatings with synergistic effects [90]. For instance, the CuO-TiO_2_ spray-coated polypropylene mentioned above was both highly hydrophobic and highly biocidal. Likewise, adding Ag or TiO_2_ nanoparticles into a rough nanostructured polymer matrix yields coatings that resist fouling and actively kill any attached microbes [96,97].

In study [95], the authors investigate how biomimetic titanium nanopillars influence bacterial physiology and morphology. They report that these nanostructures cause deformation and partial penetration of both Gram-positive and Gram-negative bacterial cell envelopes, without leading to cell rupture or lysis. Additionally, the nanopillars interfere with bacterial cell division and promote the generation of reactive oxygen species, along with an increased presence of proteins associated with oxidative stress. These findings suggest that the antibacterial effect of the nanopillars is largely driven by oxidative stress mechanisms rather than direct cell destruction.

**Figure 7:**
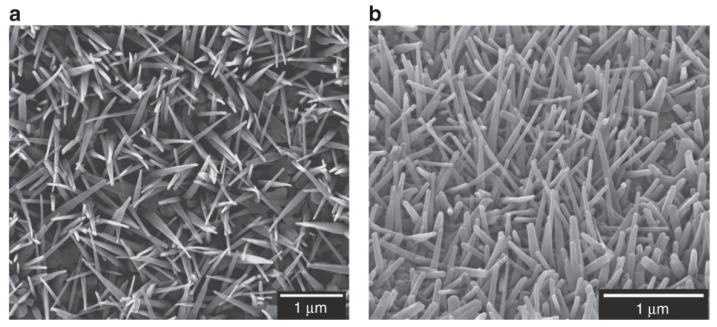
Scanning electron microscopy (SEM) images of the TiO_2_ nanopillar surface NW-850-5 are shown from (**a**) a top-down perspective and (**b**) at a 40° tilt angle. The NW-850-5 surface was produced by thermal oxidation at 850 °C for 5 min. Scale bars correspond to 1 µm. The images are representative of three independent thermal oxidation batches (*n* = 3), with each batch comprising 25 individual surfaces [95]. (The figure is available Open Access).

The nanopillars were fabricated on grade 5 titanium alloy (Ti-6Al-4V), a material commonly used in orthopedic implants, using a thermal oxidation process. Specifically, the surface referred to as NW-850-5 was produced by oxidation at 850 °C for 5 min, resulting in a nanotextured structure that closely resembles the nanoscale protrusions observed on dragonfly wings. The formed nanopillars are composed of TiO_2_, predominantly in the rutile phase. SEM images illustrating these nanostructures are shown in Figure 7.

Deformation of the cell envelope caused by nanopillars was observed in *S. aureus*, *E. coli*, and *K. pneumoniae*, and these findings were in partial agreement with the proposed contact-killing mechanism. This model proposes that when bacteria attach to nanopillar surfaces, their cell envelopes are stretched until they rupture mechanically, ultimately causing cell death. However, it remained uncertain whether this deformation actually results in penetration of the bacterial envelope—that is, whether nanopillars can puncture the envelope and compromise the integrity of the barrier separating the cytosol from the surrounding environment.

When evaluating antimicrobial coatings, typical metrics include log reduction in CFU (colony-forming units) over a fixed contact time, percent kill rate, or survival fraction (e.g., ISO 22196 for antibacterial surfaces). Many studies report multi-log reductions (e.g., 3–6 log kill) within hours of contact. For viruses, plaque assays or PCR-based viability tests are used. Researchers also assess durability of the antimicrobial function (e.g., repeated washing or wear cycles). Real-world field trials are relatively rare but illustrative: for example, a hospital installed copper-alloy rails and observed significant decreases in surface microbial burden in emergency rooms [98,99,100,101].

Beyond metals, polymer coatings with antimicrobial functionalities are emerging. Polymer brush coatings (PEG, zwitterionic, polyoxazolines) are “anti-fouling” in that they resist protein/bacterial adhesion [102,103]. While not directly biocidal, such non-fouling polymers keep surfaces essentially sterile by preventing any stable attachment. Others graft cationic polymers (quaternary ammonium, quaternized chitosan) onto surfaces; these kill microbes by electrostatic membrane disruption. Embedding enzymes or antimicrobial peptides into polymer coatings is also under study. Such organic approaches offer the advantage of low toxicity, but stability and longevity can be limiting. In all cases, a key challenge is balancing efficacy against concerns of toxicity and environmental impact. Leaching of metals (especially nanosilver) must be controlled to avoid cytotoxicity or resistance evolution [104,105,106,107].

### 2.4. Combined and Multifunctional Coatings

An important trend is the design of hybrid coatings that integrate multiple self-cleaning mechanisms. For instance, photocatalytic-superhydrophobic hybrids aim to combine water-repellency with ROS generation. A fluorinated TiO_2_ coating can both shed rainwater and kill microbes under light. In practice, this is implemented by first creating a hierarchical (micro/nano) structure and then depositing a photocatalyst. Such surfaces have been shown to keep themselves dry (preventing biofilm formation) while also oxidizing organic residue [108,109,110]. Similarly, metal-polymer composites can pair rapid contact killing with controlled release. The CuO-TiO_2_/polymer spray is one example, as are silver-coated superhydrophobic fabrics that repel fluids and inactivate bacteria on contact [111,112].

Ocean-based antifouling is another area of hybrid approaches. Superhydrophobic paints for hulls reduce adhesion of barnacles and algae, while embedded biocides (e.g., ZnO or CuO nanoparticles) kill any organisms that settle [113,114]. In marine studies, combining a nano-textured hydrophobic surface with a coating of TiO_2_ or Ag has shown marked improvements in foul-release performance. For example, one study sprayed a siloxane-based binder with Ag_3_PO_4_ nanoparticles onto PVC, yielding a coating that was both superhydrophobic and bactericidal against *E. coli* (showing near-complete growth inhibition) [70].

These multifunctional coatings are promising but also challenging to scale. Fabrication complexity increases (multiple layers or steps), and long-term stability of the combined functionalities must be demonstrated. Care must be taken that one function (e.g., hydrophobicity) is not compromised by the other (e.g., UV exposure for photocatalysis). Nonetheless, the potential payoff—surfaces that repel dirt and sterilize themselves—is driving active research. Combining contact-killing, nanostructuring, and hydrophobicity can create “super-antimicrobial” surfaces for high-touch applications [115,116].

### 2.5. Applications Across Sectors

The broad range of applications for these coatings is a key selling point. In healthcare, hospital-acquired infections drive demand for self-sanitizing surfaces. Coated door handles, bed rails, touchscreens and medical instruments can reduce pathogen loads without constant disinfection. For example, nanosilver-impregnated paints or TiO_2_ coatings have been tested in patient rooms, showing lower counts of MRSA and other hospital pathogens. In one emergency room trial, a nanosilver/titanate coating on countertops lowered microbial loads by >90% [67]. Similarly, as mentioned, copper alloy surfaces (antimicrobial on contact) are used for high-touch fittings; SARS-CoV-2 survival was far shorter on such surfaces [90].

In public spaces and transportation, self-cleaning coatings can reduce maintenance. Hydrophobic coatings on subway seats or escalator handrails can help them stay clean between cleaning cycles. Photocatalytic paints on tunnel walls degrade graffiti and pollutants under sunlight. Even consumer electronics (keyboards, phones) have seen interest in coatings that inactivate microbes by touch. During the COVID-19 pandemic, some companies developed silver- or graphene-based antiviral coatings for masks and PPE, combining filtration with surface disinfection [117,118,119,120].

Marine engineering leverages antifouling coatings on ship hulls, piers and cooling systems. Traditional antifouling relied on toxic biocidal paints (copper, tributyl tin), but self-cleaning alternatives are emerging. Hierarchical fluorinated coatings can repel water and slimy organisms (biofilm), and embedded photocatalysts or biocides can further deter growth. Superhydrophobic, anti-corrosive coatings can greatly reduce drag and biofouling in seawater. Field tests have demonstrated, for instance, that coated panels accumulate far fewer barnacles over months than bare steel panels [121,122,123,124,125].

**Figure 8:**
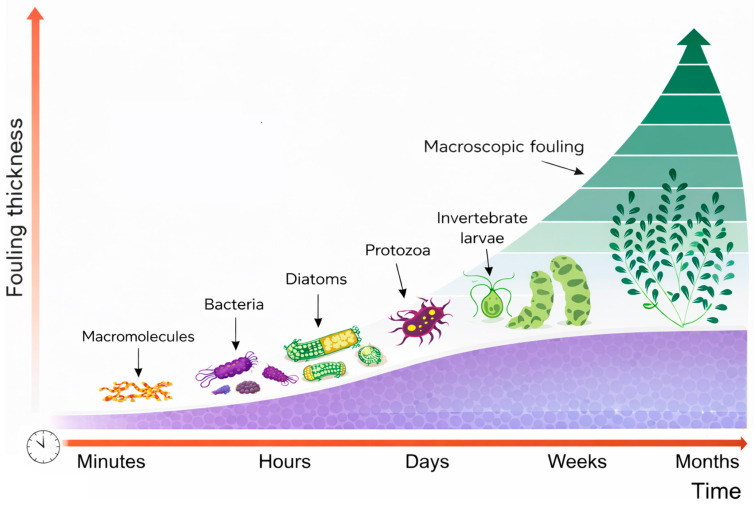
Illustration outlining the four characteristic stages of marine biofouling. Reproduced from [124]. (The figure is available Open Access).

Biofouling is a complex, multi-step phenomenon that generally progresses through four main phases, illustrated in Figure 8 initial surface conditioning and biofilm development (stages I and II), followed by microfouling (stage III) and macrofouling (stage IV). When a material is immersed in water, various organic and inorganic macromolecules-such as proteins and polysaccharides-rapidly accumulate on the surface, forming a preliminary conditioning film (stage I). Shortly thereafter, early colonizers including bacteria and algae adhere to this layer, leading to the formation of a developed biofilm (stage II). This biofilm, rich in extracellular polymeric substances produced by the organisms, creates a favorable environment for the attachment of additional species. Notably, these initial two stages are typically reversible, as microorganisms like bacteria and diatoms can still be removed relatively easily [124].

In food packaging and processing, antibacterial coatings extend shelf life. Polymers incorporating AgNPs or ZnO act as antimicrobial liners on plastic wraps or container surfaces. These coatings slowly release ions that suppress bacterial and fungal growth on food contact surfaces [74]. Many smart packaging concepts also exploit the optical properties of nanomaterials (e.g., AgNP films that change color to signal spoilage). Importantly, these food-grade coatings must be non-toxic at low residue levels; recent reviews emphasize that controlled, sustained release of silver can provide long-lasting protection against spoilage organisms [126].

Other sectors include water treatment (self-disinfecting membranes), HVAC systems (photocatalytic air filters), and construction materials (self-cleaning concrete or paints that keep walls algae-free). For instance, TiO_2_-impregnated concrete degrades organic soot on tunnels and buildings. Even consumer applications, like self-sanitizing table surfaces or doorknob covers, are under development. Each real-world use demands specific performance tests (e.g., marine coatings tested under flow, healthcare coatings under hospital lighting and humidity) [127,128,129].

### 2.6. Barrier and Protective Polymer Films

Another key functionality is creating high-performance barrier films that block gases, moisture, or chemicals. In food packaging, polymer films (e.g., polyesters, polyolefins, or bio-based polymers) are engineered for ultra-low oxygen and water vapor transmission to preserve freshness [130,131]. For instance, ethylene vinyl alcohol (EVOH) or nanocomposite coatings (clay or graphene in a polymer) can reduce O_2_ permeability by orders of magnitude. Recent work has focused on biopolymer and edible coatings: biodegradable films from starch, cellulose or proteins can incorporate lipids or nanoparticles to improve moisture resistance [132,133,134]. These edible films act as semi-permeable barriers: they seal produce with a thin layer (<0.25 mm) that limits gas exchange (oxygen, CO_2_) and moisture loss, thereby slowing oxidation and spoilage. However, natural polymers generally have poor water vapor barrier and mechanical strength, so multilayer structures or nanofillers (e.g., nano-cellulose, chitosan) are often added [135,136]. Food-grade coatings must also be safe, transparent, and sometimes antimicrobial; smart materials (e.g., phenolic additives that scavenge oxygen, or bacteriocins for antimicrobial action) are active research areas [137,138,139,140].

Active compounds are typically integrated into packaging materials or applied to their surfaces in forms such as sachets, labels, or absorbent pads. These agents can be either synthetic or naturally derived and are intended to provide functions like antimicrobial or antioxidant activity. In recent years, there has been increasing interest in natural alternatives, largely due to concerns about the possible health risks linked to synthetic additives and preservatives.

**Figure 9:**
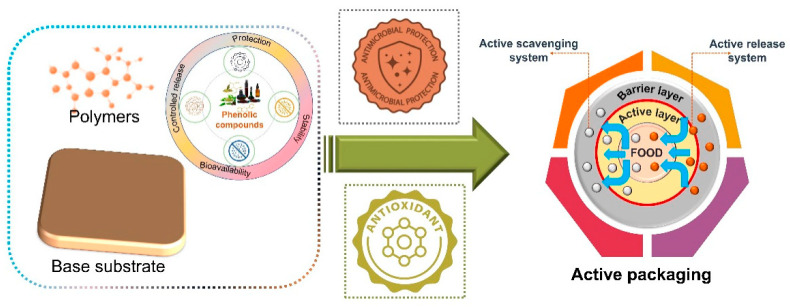
Visual representation highlighting the role of phenolic compounds in imparting antioxidant and antimicrobial functionalities within active packaging systems [137]. (The figure is available Open Access).

Many foods—including fruits, vegetables, herbs, spices, seeds, tea, chocolate, and wine-serve as rich sources of naturally occurring antioxidants and antimicrobial substances, particularly phenolic compounds. In addition, these bioactive ingredients are often obtainable from underexploited plant materials and food industry byproducts, making them attractive not only for their functional properties but also for their economic potential [137]. Figure 9 depicts an active food packaging system in which phenolic compounds are incorporated into polymer-based materials coated onto a base substrate to impart functional properties. These natural bioactive compounds provide antimicrobial and antioxidant protection while enabling controlled release and improved stability within the packaging matrix. The resulting system consists of a multilayer structure with a barrier layer and an active layer that interacts dynamically with the food through two main mechanisms: scavenging unwanted substances such as oxygen or free radicals, and releasing beneficial compounds to inhibit microbial growth and oxidation. Overall, the design transforms conventional packaging into an active system that enhances food preservation, safety, and shelf life.

In electronics and optoelectronics, barrier polymers must be transparent and flexible while blocking moisture and oxygen to protect sensitive components (OLEDs, displays, solar cells). Recent advances include multilayer polymer/oxide stacks and self-healing polymer films. For example, Mahmood et al. demonstrated fully polymeric gas-diffusion-barrier films by alternately layering 2D polymer sheets (a porous crystalline polymer) and cross-linked PDMS on PET substrates [141]. These ultrabarrier films achieved a water vapor transmission rate as low as 8.5 × 10^−4^ g·m^−2^·day^−1^—a nearly 10,000-fold reduction compared to bare PET—while remaining flexible through thousands of bends. Intriguingly, the authors [141] also applied a self-healing PDMS elastomer on the film’s backside, imparting both self-cleaning and crack-healing functionality in a roll-to-roll compatible process. Similarly, transparent ceramics and inorganic–organic hybrids (e.g., silica-polymer films) are being developed to combine optical clarity with gas blocking. These films are critical for flexible devices (e.g., foldable phones) and emerging technologies like indoor photovoltaics.

Anticorrosion coatings form another category of protective polymer films. Traditional anti-corrosion paints are multilayer organic coatings on metals: typically a primer (for adhesion), one or more functional mid-coats (for barrier or inhibitor release), and a topcoat for UV/abrasion protection [142]. Modern polymeric coatings for corrosion often incorporate smart features: microcapsules of corrosion inhibitor or dynamic covalent chemistries (vitrimers) that can seal cracks upon damage. These self-healing polymer coatings can autonomously repair micro-cracks and restore the barrier function [143,144]. By healing after scratches, such coatings can greatly extend service life and reduce maintenance costs [145,146,147]. Standard polymer barriers already dramatically lower corrosion rates: the IJCSI review notes that organic coatings “form a continuous film over the substrate to effectively block corrosive species”, and they can be engineered with nanoparticles for improved barrier properties. For example, adding nano-silica or layered silicates to an epoxy can create a tortuous path that slows diffusion of water and ions. Overall, polymeric anticorrosion coatings are indispensable in infrastructure, automotive, and marine industries, and ongoing developments aim to make them more sustainable (waterborne, low-VOC) and functional (sensors, self-healing) [148,149,150,151].

### 2.7. Advantages, Limitations

Each class of coating has trade-offs. Photocatalytic coatings offer active destruction of contaminants and are self-regenerating, but require a light source and may produce unwanted by-products. Recent work addresses these issues by developing visible-light-responsive catalysts and optimizing light absorption [152,153]. They can be transparent (suitable for windows) and non-toxic, but long-term stability (photocorrosion, deactivation by dirt) is a concern. Hydrophobic/superhydrophobic coatings provide passive, continuous self-cleaning without chemicals; however, they generally cannot kill pathogens and rely on surface roughness, which can be mechanically fragile. Durability under abrasion or UV light is often poor for superhydrophobic surfaces, and fluorinated chemistries raise environmental concerns. Ongoing research on bio-based low-energy coatings aims to overcome these limitations [154,155].

Antimicrobial coatings with biocides (metals, QACs, etc.) are potent and simple to use, but there are safety and ecological issues. Leaching of silver or copper can be cytotoxic at high doses and can contribute to microbial resistance. Regulatory approval for biocidal surfaces (especially antiviral claims) can be challenging [156,157]. Furthermore, continuous killing action may disrupt beneficial microbiomes if surfaces contact skin or food. Thus, the most promising designs control release rates and use non-toxic concentrations. Nanotextured surfaces that kill mechanically avoid chemicals but must be proven non-hazardous and cost-effective at scale [158,159,160].

Future research will likely emphasize multifunctional coatings that integrate several mechanisms for synergistic effect. For example, combining hydrophilic self-cleaning layers with hydrophobic anti-adhesion layers, or pairing photocatalysis with superhydrophobicity as described above. Improved fabrication techniques (e.g., layer-by-layer assembly, 3D printing of composites) will enable complex architectures [161,162]. Emerging materials—graphene and other 2D materials, metal–organic frameworks (MOFs) that release gases (Cl_2_, NO), enzyme-mimetic nanoparticles—are being explored to add functionalities. Sustainability is also a driver: replacing perfluorinated compounds and finding biodegradable coating matrices are urgent goals [163,164].

In summary, the last decade has seen an explosion of novel coatings for surface hygiene. Photocatalytic films, bioinspired hydrophobic textures, and advanced antimicrobial composites each offer compelling advantages. A balanced view acknowledges that no single coating is ideal for all situations. Instead, the “best” solution often combines mechanisms tailored to the application’s environment and threat profile. With careful design, testing, and real-world trials, these emerging coatings hold great promise to reduce contamination, biofouling and infection risk across many industries [165,166,167,168,169].

## 3. Plasma-Based Strategies for Polymer Surface Modification

Polymer surfaces are inherently hydrophobic and chemically inert, exhibiting low surface energy and poor adhesion. These characteristics hinder applications requiring strong bonding, wettability, or biological compatibility. Traditional modification methods (wet chemistry, flame, corona) often involve solvents or high temperatures. In contrast, cold (nonthermal) plasmas provide a dry, solvent-free way to activate or coat polymer surfaces [170,171,172]. Cold plasma consists of reactive ions, radicals, and UV photons that can etch, crosslink, and functionalize only the top few nanometers of a polymer without bulk damage. For example, oxygen or water-vapor plasmas efficiently introduce -OH and -COOH groups on hydrocarbon polymers, converting a hydrophobic surface to highly hydrophilic [173,174,175,176]. In plasma-enhanced chemical vapor deposition (PECVD), gas-phase monomers are ionized and polymerize on the substrate, yielding pinhole-free, conformal thin films. Thus, plasmas can tune wettability, adhesion, biocompatibility and other surface properties by controlling processes such as etching, roughening, crosslinking, and chemical grafting [177,178,179,180].

**Figure 10:**
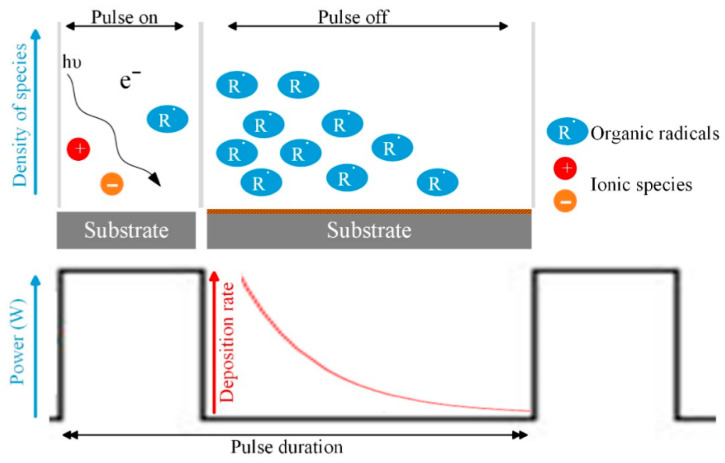
Schematic illustration showing the mechanism of polymer film formation during the pulse-on and pulse-off phases in pulsed plasma polymerization [171]. (The figure is available Open Access).

Pulsed plasma polymerization enables precise adjustment of power over time scales ranging from microseconds to milliseconds, allowing effective control of the overall power density. The process operates through two alternating phases [171]. During the pulse on-time (t_pulse-on_), monomer molecules undergo fragmentation and recombination, leading to polymer formation under conditions governed by monomer flow rate, pressure, and plasma power, similar to continuous-wave (CW) operation. In contrast, during the pulse off-time (t_pulse-off_), reactive species generated in the on-phase interact with monomers to drive further polymerization, as illustrated in Figure 10. In the absence of ion bombardment and photon irradiation, these radicals promote a predominantly chain-growth reaction mechanism. As a result, the material formed during the off-phase closely resembles conventional polymers in its chemical structure. Overall, the initiation of the reaction primarily occurs during t_pulse-on_, while chain propagation dominates during t_pulse-off_.

Plasma polymerization stands out as a versatile route to form uniform, pinhole-free polymer films with high cross-link density. In a typical process, gaseous monomers (e.g., silanes, acrylics, olefins) are fragmented into radicals and ions by high-energy electrons; these reactive species then recombine on the substrate to build a thin polymer network [181,182]. Unlike conventional solution polymerization, plasma deposition inherently yields a highly cross-linked structure due to continual fragmentation and random recombination [183]. This extreme cross-linking confers mechanical robustness and chemical stability to plasma-polymerized films, making them exceptionally durable and solvent-resistant. By choosing appropriate precursors, one can tune the surface chemistry: for example, silane monomers like tetra-methylsilane (TMS) yield hydrophobic SiO-rich coatings, while acrylic monomers generate polar hydrophilic films [184,185]. Indeed, atmospheric-pressure plasma techniques (dielectric barrier discharges, plasma jets) have been used to deposit coatings ranging from hydrophilic acrylic films to fluorinated repellent layers [186,187]. Such plasma–polymer films have been explored for antifouling applications as well: recent studies show plasma-deposited layers (e.g., from natural compounds like carvacrol) can inhibit bacterial growth and biofilm formation on surfaces [188,189,190].

Beyond plasma polymerization, other plasma treatments (e.g., inert gas or N2 plasma) can etch and roughen polymer surfaces or directly graft functional groups. For example, helium plasma has been shown to dramatically improve PTFE wettability, dropping contact angle from ~110° to ~40° after seconds of exposure [191,192]. Similarly, argon or oxygen plasmas on polyethylene or polypropylene introduce radicals and oxygen functionalities, enabling paints or glues to adhere much better [193]. Overall, plasma-based modification is a dry, environmentally benign approach that can uniformly tune adhesion and wetting without liquid chemicals [194,195,196].

### 3.1. Mechanisms of Plasma-Polymer Interaction

Cold plasmas contain energetic species (ions, electrons, excited molecules, UV) that impact polymers at ambient temperatures. When a polymer is exposed to plasma, energetic species break chemical bonds (etching or ablation) and generate free radicals on the surface [197,198]. These free radicals can then either recombine with other radicals or react with plasma-generated species to form new crosslinks or functional groups. For instance, plasma-induced free radicals on polyethylene or polystyrene chains can bond with oxygen to form surface -OH, C=O, and COOH groups [199,200]. In nitrogen plasmas, active N atoms or ions graft amine and imine functionalities onto polymers while largely avoiding over-oxidation [9]. Other gases yield different chemistry: H_2_ plasmas preferentially roughen polyethylene surfaces via hydrogen abstraction, whereas water-vapor/Ar plasmas generate OH radicals that etch and hydroxylate surfaces (e.g., roughening PMMA and introducing -O-C=O- groups) [72,201]. Fluorocarbon plasmas (CF_4_, C_2_F_4_, etc.) can either deposit hydrophobic fluorocarbon films or etch organic materials depending on the F/C ratio and bias [202,203].

Two classical models describe plasma polymer film growth: Yasuda’s Rapid Step-Growth Polymerization (RSGP) and Competitive Ablation-Polymerization (CAP) mechanisms [204]. In the plasma-induced (substrate) path, monomer molecules (with C=C or other reactive bonds) are activated on the surface by plasma species, creating radicals that propagate polymerization in situ. Alternatively, the plasma-state (gas-phase) path involves fragmentation of precursors into polymerizable intermediates (radicals, oligomers) that then deposit on the substrate. In practice, both routes can occur simultaneously, and ion bombardment or VUV photons may also etch the growing film, establishing an equilibrium between deposition and removal. The net result is a highly crosslinked, amorphous “plasma polymer” film that can incorporate tailored chemistries by choice of gas, power, and substrate bias [205,206,207,208].

### 3.2. Plasma Processing Techniques

A wide variety of plasma sources are used for polymer treatment. Low-pressure (vacuum) plasmas are common in research and microelectronics, including RF capacitive or inductive discharges and microwave plasmas. These allow uniform treatment in controlled environments, but require vacuum chambers and batch processing. Atmospheric-pressure plasmas have grown rapidly, enabling inline or large-area processing. Examples include dielectric barrier discharges (DBD), corona discharges, plasma jets, and even piezoelectric “plasma pencils”. Atmospheric DBD and jet plasmas can operate in ambient air or inert gas, modifying surfaces without vacuum systems [12,209,210,211]. For example, Dielectric Barrier Discharge (DBD) devices create a homogeneous plasma between electrodes, whereas plasma jets direct a confined plasma plume onto the polymer. These sources allow flexible treatment of heat-sensitive substrates and patterned processing. Advanced systems can even integrate a cold plasma jet into a 3D printer, treating the polymer layer just after deposition to improve adhesion for printed parts [212,213,214,215,216].

Key process parameters include power, pressure, gas composition, flow rate, substrate temperature, and treatment time. Control of these enables fine-tuning of surface functionality. Pulsed plasmas (switching RF on/off) can favor retention of certain functional groups (e.g., preserve -NH_2_ content in plasma polymer films) [200,204]. Hybrid techniques pair plasma with UV irradiation or liquid precursors. For instance, an atmospheric He plasma jet injecting an organosilane vapor (APTES) will deposit a SiOxNy-like polymer film rich in amine groups. In another example, plasma activation of poly(lactic acid) followed by UV-induced graft polymerization formed a hydrogel coating on 3D-printed PLA [20]. Such plasma-assisted grafting combines surface radicals from plasma with subsequent solution or UV polymerization [217].

### 3.3. Plasma Polymerization and Grafting

Plasma polymerization refers to forming a polymeric film by exposing monomer vapors to plasma. Unlike conventional polymerization, plasma polymers are crosslinked, have a random structure, and adhere strongly to substrates. They are extremely uniform and pinhole-free, enabling conformal coatings on complex 3D geometries [218,219]. Because the process is one-step and solventless, the chemical nature of the deposited film can be tuned by precursor choice and plasma settings [220]. For example, pulsing an acrylic monomer plasma can yield coatings with pendant hydrophilic groups, and introducing maleic anhydride in a hydrocarbon plasma produces copolymer films suitable for embedding antibiotics in wound dressings. Silane precursors (HMDSO) in plasma produce PDMS-like films that render surfaces hydrophobic or biocompatible [204].

Plasma-induced grafting is another strategy: first, the polymer surface is activated by a brief plasma exposure to create radicals or functional groups; then monomers are polymerized onto these sites in a second step. For instance, a polyethylene substrate activated by Ar plasma can be subsequently coated by polymerizing an acrylic monomer, forming a grafted interlayer that improves adhesion of paints or adhesives. Similarly, cold plasma can locally create initiators for “living” polymerization: an oxygen plasma can generate peroxide groups that later initiate radical polymer growth when exposed to acrylic monomers under mild conditions [221,222].

Both plasma polymer coatings and plasma-grafted layers are typically only tens to hundreds of nanometers thick, which is sufficient for surface functionality without bulk alteration. To build thicker layers or multilayered structures, plasma processes can be combined with conventional coatings, or deposited sequentially using different precursors. In all cases, the key advantages remain: intimate adhesion to substrate, absence of solvents, and the ability to deposit at room temperature [223,224,225].

### 3.4. Applications in Biomedical, Packaging, Electronics, and Coatings

Plasma surface treatments enable innovations across diverse fields. Major application areas include the following:

- Biomedical devices and implants. Plasma-treated polymer implants and scaffolds show enhanced cell adhesion, growth, and biocompatibility. For example, polylactic acid scaffolds exposed to nitrogen or oxygen plasmas promoted fibroblast adhesion and proliferation [226,227]. Antimicrobial polymer coatings can be fabricated by plasma polymerizing monomers with bactericidal groups (e.g., quaternary amines), and plasma sterilization (via surface radicals and ozone) improves device hygiene. Cold plasma jets have also been used to deposit bioactive thin films (collagen-like, hyaluronic acid-like) for drug delivery or tissue healing [217].
- Food and packaging. Plasma engineering of biodegradable films (e.g., starch-, cellulose-, or protein-based) can dramatically improve their properties. For instance, O_2_ or Ar plasma etches and introduces polar groups into polymer films, increasing surface energy and promoting stronger adhesion of barrier coatings or inks [228,229,230]. These treatments also roughen the surface, improving wettability and mechanical interlocking. Notably, cold plasma can integrate antimicrobial agents into packaging: UV-activated plasma processes or plasma-grafted silver nanoparticles yield films that inhibit foodborne pathogens. Plasma processing is especially valuable for modifying heat-sensitive bio-based packaging, as it leaves the bulk material intact [231,232,233,234].

In [228], the authors employed dielectric barrier discharge (DBD) plasma treatment, varying both the exposure time and excitation frequency. They found that the optimal frequency differed depending on the material: starch films performed best at 50 Hz, whereas gelatin and bacterial cellulose films required a higher frequency of 900 Hz. This highlights the strong influence of excitation frequency, a parameter that is often overlooked. Overall, plasma treatment enhanced the films’ hydrophobicity, surface characteristics, resistance to water, and mechanical performance, without the need for additional chemical or biological modifiers.

**Figure 11:**
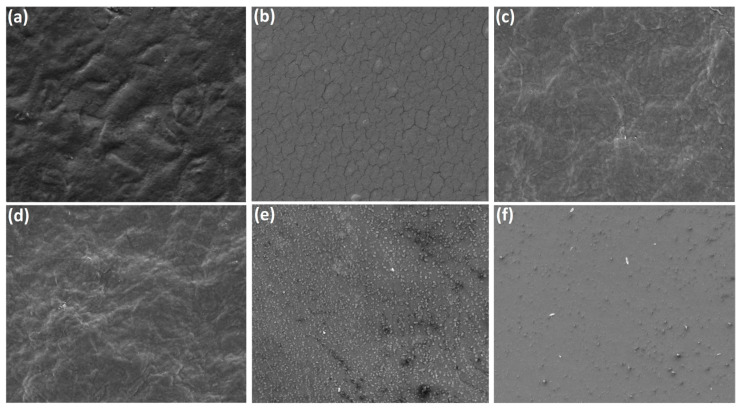
Scanning electron microscopy (SEM) images of: (**a**) untreated starch film; (**b**) starch film after plasma treatment (20 min at 50 Hz); (**c**) untreated bacterial cellulose film; (**d**) plasma-treated bacterial cellulose film (20 min at 900 Hz); (**e**) untreated gelatin film; and (**f**) gelatin film following plasma exposure (20 min at 900 Hz) [228]. (The figure is available Open Access).

For starch-based films, the untreated surface appeared rough but free of cracks (Figure 11). After plasma exposure, noticeable changes occurred: the surface became smoother, yet numerous fine cracks developed. Additionally, small white specks were observed across the surface, likely associated with a higher concentration of insoluble components within the film.

- Electronics and microelectronics. Plasma coatings are widely used for conformal protective films on printed circuit boards and components. Plasma-polymerized fluorocarbons, for example, provide pinhole-free moisture barriers that prevent corrosion on solder joints [235,236]. In microelectronics manufacturing, dielectric plasmas clean and activate polymer photoresist or polyimide layers to improve lithography and bonding. Plasma-deposited silicon- or carbon-based films serve as low-k dielectrics or hard masks [219,228]. Flexible electronics (e.g., polymer sensors, OLED displays) benefit from plasma treatments that tailor surface energy for printed metal inks or organic coatings. Plasma functionalization can also render surfaces antistatic or fluorinated for insulating layers [237,238,239].
- Functional coatings (miscellaneous). In textiles, plasma treatments impart durable water-repellency or dyeability without wet processes. For example, a plasma-polymerized fluorosilane film can make polyester fabrics superhydrophobic for waterproof clothing. Membranes for water purification and gas separation are often plasma-functionalized to increase hydrophilicity or charge, enhancing flux and selectivity [240,241,242,243]. Plasma-deposited hydrophobic coatings on filters can prevent biofouling. In the automotive and aerospace industries, plasma-polymer films (e.g., amine-, hydrocarbon-, or fluorocarbon-based) are applied as hard, ultra-thin coatings to improve wear resistance, chemical stability, or fuel-line compatibility. In each case, plasma provides a clean, conformal deposition that can be applied to complex geometries (e.g., engine components, inside pipes) [244,245,246,247].

### 3.5. Advantages, Limitations, and Comparative Scope

Advantages: Plasma treatments offer several unique benefits. They are solvent-free and typically fast (seconds to minutes) [248], making them environmentally benign and amenable to high-throughput processing. Because plasmas deposit films uniformly on all exposed surfaces, they yield highly conformal and pinhole-free coatings even on rough or three-dimensional substrates. The chemistry is highly tunable: by changing the feed gas (O_2_, N_2_, C_2_H_4_, HMDSO, etc.), one can introduce desired functional groups or bulk film chemistries [249,250,251,252]. Cold plasma leaves the polymer bulk unaffected (low gas temperatures), which is critical for temperature-sensitive materials. The plasma environment can even drive advanced phenomena such as in situ nanoparticle generation or crosslinking without additives. Overall, plasma is a “green,” tunable platform for surface engineering [219,251,253,254].

Limitations: Despite its versatility, plasma processing has constraints. The modification is generally limited to the near-surface (a few nm to 100 nm), so bulk properties (e.g., flexibility, permeability) remain dominated by the original polymer. Over-treatment can cause chain scission or discoloration. Achieving perfectly uniform treatment on large areas or non-flat parts can be challenging; atmospheric jets especially may produce spatial gradients. Scalability can be an issue: vacuum systems have limited throughput, while atmospheric units must manage factors like electrode lifetime and ozone generation [255,256,257]. Furthermore, plasma chemistries are complex and material-specific, so process optimization is often required for each polymer and desired outcome. Finally, the initial equipment cost for RF/microwave or high-frequency plasma sources can be higher than simple chemical methods. Nevertheless, ongoing advances (see below) are steadily mitigating these issues [209,254,258].

### 3.6. Future Trends

In recent years the field has seen several important trends. Atmospheric and in-line processing have expanded with roll-to-roll plasma units and handheld jets, allowing treatment of continuous films or large components. Cold plasma sources are increasingly integrated into manufacturing equipment (for example, a plasma jet coupled to a 3D printer [217]) and additive fabrication workflows. Precursor innovation is another frontier: bio-based monomers and novel organics are being plasma-polymerized to produce sustainable coatings or stimuli-responsive films. The use of plasmas to create self-healing or functional composites is emerging—for example, embedding functional nanoparticles in situ during plasma polymerization to yield healing agents or sensors. Microscale and selective plasmas (e.g., microplasma jets, mask-patterned discharges) are enabling localized surface patterning for microfluidics and lab-on-chip devices [251,259,260,261].

From a fundamental standpoint, new diagnostic tools (e.g., in situ optical emission actinometry) and models (improved CAP/RSGP theories) are improving control over plasma polymer chemistry. Researchers are also combining plasma with other methods—such as plasma-enhanced atomic layer deposition or plasma-assisted 3D printing—to create hybrid materials. As a result, we can expect plasma technologies to become even more versatile for polymers: enabling biofunctional surfaces for medical implants, smart packaging with integrated sensors, and next-generation electronics with atomic-level interface control. In summary, cold plasma and plasma polymerization remain powerful strategies for tailoring polymer surfaces, and ongoing developments continue to broaden their applications [197,204,262,263,264,265,266].

## 4. Discussion

The rapid development of functional polymer films and coatings over the past decade reflects a clear shift from passive protection toward multifunctional and responsive surface systems. As highlighted throughout this review, advances in material design, nanostructuring, and surface engineering have enabled coatings that not only act as barriers but also actively interact with their environment. This transition is particularly evident in the emergence of self-cleaning, antimicrobial, and plasma-engineered coatings, which address increasingly complex industrial and societal demands [19,20,21,22,81].

One of the key observations from the reviewed literature is that no single coating strategy is universally optimal. Photocatalytic coatings, for example, offer highly effective degradation of organic contaminants and strong antimicrobial performance through the generation of reactive oxygen species [38,39,40,41]. However, their reliance on light activation-especially UV radiation in the case of TiO_2_-limits their efficiency in indoor or low-light environments [55,56]. Efforts to extend photocatalytic activity into the visible spectrum through doping and heterostructure design show promise, but challenges related to long-term stability and potential by-product formation remain [44,45,46,64]. In contrast, hydrophobic and superhydrophobic coatings provide a passive, energy-independent self-cleaning mechanism based on the lotus effect, effectively reducing surface contamination and biofouling [23,24,67]. Yet, their inability to actively kill microorganisms and concerns regarding mechanical durability and environmental impact (e.g., fluorinated compounds) constrain their broader application [78,79,80].

Active antimicrobial coatings address some of these limitations by directly inactivating pathogens through biocidal mechanisms. Metal-based systems, particularly those incorporating silver, copper, or zinc, demonstrate high antimicrobial efficacy and have already found widespread application in healthcare and public environments [89,90,91,92,93,94]. Nevertheless, issues such as ion leaching, cytotoxicity, and the potential development of microbial resistance necessitate careful material design and regulation [104,105,106,107]. Contact-active and nanostructured surfaces provide an alternative approach by avoiding the release of biocides, instead relying on surface chemistry or physical interactions to disrupt microbial cells [85,86,87,88,95]. While promising, these systems often face challenges related to scalability, durability, and consistent performance under real-world conditions.

A significant trend identified in this review is the increasing focus on hybrid and multifunctional coatings. By combining multiple mechanisms—such as photocatalysis with superhydrophobicity or antimicrobial agents with anti-adhesive surfaces-researchers aim to overcome the limitations of individual approaches and achieve synergistic effects [108,109,110,115]. These multifunctional systems are particularly relevant for high-risk environments, such as hospitals or marine applications, where both contamination prevention and active sterilization are required. However, integrating multiple functionalities into a single coating introduces additional complexity in fabrication, cost, and long-term stability, highlighting the need for optimized design strategies and scalable production methods.

Plasma-based surface modification emerges as a unifying and enabling technology across many of these applications. Its ability to tailor surface chemistry, improve adhesion, and deposit uniform, pinhole-free films without the use of solvents makes it highly attractive from both a performance and sustainability perspective [170,171,172,219]. Plasma processes also facilitate the incorporation of functional groups and nanostructures that are difficult to achieve by conventional methods, supporting the development of advanced coatings with controlled wettability, biocompatibility, and barrier properties [177,178,179,180]. Despite these advantages, limitations related to equipment cost, process scalability, and surface-only modification depth must be considered when translating laboratory results into industrial practice [255,256,257].

In the context of barrier and protective films, the reviewed studies demonstrate substantial progress in enhancing resistance to gases, moisture, and chemical agents, particularly for food packaging and flexible electronics [130,131,132,133,134,141]. The integration of nanofillers and multilayer architectures has significantly improved barrier performance, while emerging self-healing and biodegradable systems address sustainability concerns [143,144,145,146,147]. However, balancing mechanical strength, transparency, and environmental compatibility remains a key challenge, especially for next-generation applications requiring flexible and eco-friendly materials.

Overall, the literature indicates that future advancements in polymer coatings will likely be driven by the convergence of multiple disciplines, including materials science, surface physics, and nanotechnology. Emphasis is expected to shift toward sustainable materials, fluorine-free chemistries, and energy-efficient fabrication techniques, alongside the continued development of multifunctional and adaptive surfaces [163,164]. While significant progress has been made, further research is needed to address durability, large-scale manufacturing, and real-world performance validation. Bridging the gap between laboratory innovation and industrial implementation will be critical to fully realizing the potential of advanced polymer coating technologies.

In conclusion, polymer films and coatings have evolved into highly versatile and indispensable materials across a wide range of industries, including healthcare, packaging, electronics, and environmental engineering. This review has highlighted the significant progress made in designing functional coatings that go beyond traditional protective roles, offering advanced properties such as self-cleaning, antimicrobial activity, and enhanced barrier performance. These developments are largely driven by the need to address modern challenges related to contamination control, material durability, and sustainability.

Among the various approaches discussed, photocatalytic, hydrophobic, and antimicrobial coatings each provide distinct advantages, though they also present specific limitations that must be carefully considered in practical applications. Increasingly, research efforts are focused on combining these functionalities into hybrid systems capable of delivering synergistic performance. In parallel, plasma-based surface modification has emerged as a powerful and environmentally friendly tool, enabling precise control of surface chemistry and the fabrication of uniform, high-performance coatings without the use of harmful solvents.

Despite these advances, several challenges remain, particularly in terms of long-term durability, large-scale manufacturing, and environmental impact. Future research is expected to prioritize sustainable materials, fluorine-free chemistries, and multifunctional designs that maintain performance under real-world conditions. Overall, continued innovation in polymer coating technologies holds strong potential to improve material performance, reduce environmental footprint, and contribute to safer and more efficient systems across diverse applications.

## Data Availability

No new data were created or analyzed in this study. Data sharing is not applicable to this article.
